# Quantifying compliance and acceptance through public and private social conformity

**DOI:** 10.1016/j.concog.2018.08.009

**Published:** 2018-10

**Authors:** Sophie Sowden, Sofia Koletsi, Eva Lymberopoulos, Elisabeta Militaru, Caroline Catmur, Geoffrey Bird

**Affiliations:** a Social, Genetic and Developmental Psychiatry Centre, Institute of Psychiatry, Psychology and Neuroscience, King’s College London, London SE5 8RJ, UK; b Department of Psychology, Institute of Psychiatry, Psychology and Neuroscience, King’s College London, London SE5 8RJ, UK; c Department of Experimental Psychology, University of Oxford, Oxford OX1 3PH, UK

**Keywords:** Compliance, Acceptance, Social conformity, Normative influence, Informational influence

## Abstract

Social conformity is a class of social influence whereby exposure to the attitudes and beliefs of a group causes an individual to alter their own attitudes and beliefs towards those of the group. Compliance and acceptance are varieties of social influence distinguished on the basis of the attitude change brought about. Compliance involves public, but not private conformity, while acceptance occurs when group norms are internalised and conformity is demonstrated both in public and in private. Most contemporary paradigms measuring conformity conflate compliance and acceptance, while the few studies to have addressed this issue have done so using between-subjects designs, decreasing their sensitivity. Here we present a novel task which measures compliance and acceptance on a within-subjects basis. Data from a small sample reveal that compliance and acceptance can co-occur, that compliance is increased with an increasing majority, and demonstrate the usefulness of the task for future studies of conformity.

## Highlights

• Most measures of social conformity conflate compliance and acceptance.
• Compliance occurs when individuals conform in public, but not in private.
• Acceptance occurs when group influence is internalised, in private and in public.
• Our task reveals the presence of compliance and acceptance on a within-subject basis.
• The magnitude of compliance increases as the size of the majority increases.

## 1 Introduction

The multitude of ways in which the behaviour and attitudes of others impact our own has been studied since the very inception of psychology (Cialdini & Goldstein, 2004). A particular focus has been the study of how groups influence the behaviour of the individual, with studies such as those of Asch on social conformity (Asch, 1951, Asch, 1955, Asch, 1956) some of the most well-known in the field. In these studies participants were asked to complete a simple perceptual task (judging the length of lines) in a group setting where, unbeknownst to the participant, the other members of the group were confederates of the experimenter. During critical trials, despite the task having an obvious answer, the confederates all gave the incorrect answer. Only a quarter of participants remained completely independent of the group, with the rest showing various degrees of conformity towards the group’s responses.

Subsequent work has identified various types of group influence, individuated by factors including the circumstances of the influence (e.g. whether the group pressure is explicit or implicit), and the nature of the change brought about in the individual. With respect to the latter, of interest to the current study is the distinction between compliance and acceptance (Kelman, 1958, Nail et al., 2013). Compliance and acceptance can be distinguished based on the type of attitude change brought about by the social influence. Compliance occurs when the individual publicly agrees with the group but does not change their own attitude or belief, whereas acceptance occurs when the social influence causes the individual to internalise the belief or attitude expressed by the group such that it becomes their own. Compliance and acceptance are thought to arise primarily from normative and informational influence, respectively (Abrams et al., 1990, Deutsch and Gerard, 1955). Normative influence occurs due to the desire of individuals to be accepted by the group, or at least not to be publicly in conflict with the group. Abrams et al. (1990, page 98) suggest that “compliance with the demands and expectations of other group members and overt agreement with their views occur because of their power to reward, punish, accept or reject individual members.” In contrast, informational influence is thought to result in acceptance because it occurs when individuals look to others for evidence as to the state of the world. As such, its effects are thought to be maximal when the state of the world is ambiguous, or when the individual is uncertain about a decision or judgement (Cialdini and Goldstein, 2004, Cialdini and Trost, 1998, Deutsch and Gerard, 1955). Thus, both informational and normative influence may reduce conflict between beliefs held by the self and those received from others. However, normative influence involves the reduction of *public* conflict with others in a group, whereas informational influence results in a reduction of conflict between incompatible beliefs within the individual.

Although it is commonly accepted that both types of social influence are typical in everyday social situations, social conformity effects obtained using the Asch paradigm are usually attributed to normative influence (compliance) only (e.g. Allen, 1965, Allen, 1975, Bond and Smith, 1996, Turner, 1991). The fact that the perceptual decision task has an obviously correct answer (participants almost never make errors on trials where confederates give the correct answer) is usually taken as *prima facie* evidence that results occur due to compliance to the group decision through normative influence. This view is not universally accepted however (e.g. Abrams et al., 1990, Turner, 1985), and claims of an informational influence are supported by a handful of studies that have compared levels of conformity using this task between groups of individuals who must respond publicly, and those who have the opportunity to make their responses in private.

**Fig. 1:**
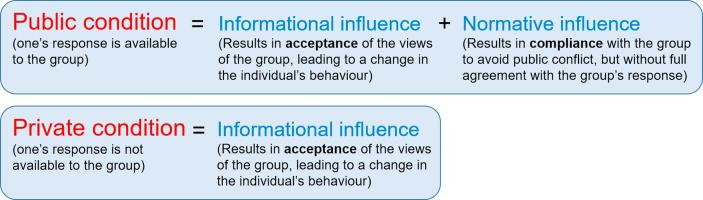
Diagram to define key terms and their relationship to one another.

The logic of these experiments is that, by comparing individuals who give their responses in public with those who respond in private, the relative contributions of normative and informational influence (and hence compliance versus acceptance) can be established (see Fig. 1). Individuals who respond in private should experience little to no normative influence due to the fact that the group members are unaware if they have conformed or not, and thus any group influence should be due to informational influence alone. Comparison of the degree of social conformity in private and public groups therefore allows the extent of normative and informational influence to be established using the standard Asch paradigm.

Results of studies which have compared public and private conformity effects support the existence of an informational effect in the Asch paradigm as well as a normative effect. For example, Asch (1956; Experiment 4) found that rates of conformity (the percentage of critical trials across all participants in which errors in the direction of the confederates’ judgements were made) dropped from 43% in public conditions to 12.5% in private conditions – demonstrating a substantial normative influence effect. However, the 12.5% conformity rate in the private conformity condition was higher than the 1% error rate observed in control groups who were not subject to group pressure to give incorrect answers. This indicates the presence of an informational influence, albeit of smaller magnitude than the normative influence. Abrams et al. (1990) reached a similar conclusion, noting that participants conformed on an average of 58% of trials when asked to respond publicly, but only 33% when responding privately (see also Deutsch & Gerard, 1955 [comparison of Experiment 1: face-to-face condition and Experiment 2: anonymous, no commitment condition], where they reported a 16% drop in conformity using a privacy manipulation).

While it is logically coherent to compare public and private responses to identify the relative contribution of normative and informational influence in the Asch paradigm, the current implementation of this comparison can be improved. Thus far, the manipulation of public and private responses has been between groups: the public group make their responses as per the original paradigm, whereas in the private group, although the confederates give their responses publicly, the participant responds in private. Groups of participants who respond publicly are then compared with those who respond in private. One issue with this approach is that comparisons between groups have the potential to be affected by sampling error and do not account for subject-level variance; as is typical therefore it is likely that within-subjects comparisons would be more sensitive than between-groups comparisons. The loss of sensitivity with between-subjects comparisons is especially important when using techniques with a low signal-to-noise ratio such as functional Magnetic Resonance Imaging (fMRI), which have been used several times to examine the neural correlates of conformity-related processes (Berns et al., 2005, Campbell-Meiklejohn et al., 2012a, Klucharev et al., 2009, Klucharev et al., 2011). Perhaps more important is the potential confounding effect of asking participants to respond in private while the confederates, who the participant believes to be other participants, respond publicly. Abrams and colleagues have argued that the distinction between the way in which the group of confederates respond (publicly), and the way in which the participant responds (privately), may result in the participant feeling like an out-group member. This may cause them to anti-conform to the confederates, reducing the observed magnitude of any conformity effect due to informational influence. Ideally then, the private / public response manipulation would be on a within-subjects level and the participants and confederates would respond in a similar manner.

The Asch task itself has been criticised on a number of methodological grounds, several of which were noted soon after the Asch experiments were originally published (e.g. Crutchfield, 1955). Chief among these criticisms is the fact that the original Asch task is an insensitive measure; the choice of only three response options, two of which are very obviously wrong, presumably means that a great deal of pressure to conform must be experienced before an incorrect option is chosen. Small effects of social influence are therefore likely to go undetected. The use of only three response options also means the size of any conformity effect is impossible to measure; therefore, the degree of conformity in the task refers to the frequency of conformity across trials, rather than the size of the effect on any one trial. The use of a continuous response scale would alleviate these problems, although as far as we are aware continuous response scales have only been used with ‘off-line’ conformity paradigms where participants do not interact directly with group members in real life, but instead receive false feedback as to the responses of a group who had previously completed the task (e.g. Campbell-Meiklejohn et al., 2012a, Campbell-Meiklejohn et al., 2012b, Klucharev et al., 2009, Klucharev et al., 2011, Zaki et al., 2011). Meta-analytic work has demonstrated that off-line conformity paradigms result in reduced conformity effects compared to on-line paradigms in which participants interact with group members, and off-line paradigms are thought to induce conformity through different mechanisms to on-line paradigms (Bond and Smith, 1996, Deutsch and Gerard, 1955, Levy, 1960).

This paper therefore reports data obtained from a relatively small sample of participants primarily to illustrate a novel on-line social conformity task based on the original Asch paradigm. The task was designed to measure the effects of normative and informational influence on a within-subjects level utilising public and private responses – thus identifying whether both compliance and acceptance may be induced in individuals by the group. Furthermore, participant and confederate responses were obtained using the same method, eliminating a factor which may have resulted in the participant feeling like an out-group member and anti-conforming from group responses in previous studies. Finally, participants were able to use a continuous response scale, meaning that small effects of social influence could be detected.

Participants were asked to take part in a study on ‘the effect of motor preparation on perception.’ They were told that they would be tested in groups of four, for efficiency, and asked to judge the colour of a patch on a central screen. They were informed that they would need to type their responses on some trials, and speak their responses on other trials as the experiment was designed to compare the effect of preparing a manual versus a vocal response on colour perception. The order of responding was fixed such that the participant was the last to give a response. Thus, randomly across trials, it was either the case that the participant and confederates all typed their responses (baseline trials in which no social influence was present), the participant typed their response after the confederates had spoken their responses out loud (private conformity condition), the participant and confederates gave spoken responses (public conformity condition), or the participant and two of the three confederates gave a spoken response while one confederate typed their response (‘reduced majority’ trials in which the participant responded publicly but where the majority consisted of 2 rather than 3 confederates). Reduced majority trials were also included to ensure the response style for confederates and participants did not differ across trials (i.e. all participants experienced private trials).

Given previous results (Abrams et al., 1990, Asch, 1956, Deutsch and Gerard, 1955), three main predictions can be made. First, it was predicted that conformity would be observed during both public and private conditions, as indexed by the difference in participants’ responses between trials in which the confederate responses were congruent (i.e. accurate) and incongruent (i.e. inaccurate) with respect to the correct response. Second, it was hypothesised that both acceptance and compliance can be induced in the same individual to bring about conformity. Acceptance and compliance can be identified, respectively, by an informational influence effect (indicated by private conformity) as well as a normative influence effect (indicated by greater public compared to private conformity). Third, it was predicted that the normative effect (compliance) would be greater in magnitude as the size of the majority increased from 2 to 3 confederates (Asch, 1955). The presence of reduced majority trials, where only 2 confederate responses are available (compared to 3 on a standard public trial) allow for this comparison to be made.

## 2 Materials and methods

### 2.1 Participants and design

Twenty-two healthy adult female individuals (mean age = 21.2 years, SD = 2.8) were recruited via the King’s College London research recruitment website. Thus, all participants were either staff or students at King’s College London, from a wide variety of disciplines. For recruitment to the study, it was a requirement that participants had not previously studied psychology at college or higher education level. Only female participants were recruited in order to remove the potential for out-group effects based on the sex of the participant compared to that of the (female) confederates. Participants were informed that this was a study investigating the effect of ‘motor preparation on perception’ and that the investigators were interested in better understanding how motor preparation for spoken versus typed responses affects visual perception. They were also told that to speed up data collection, and depending on the number of participants signed up to the timeslot, they would be tested in a group of up to 4 participants. In fact, each participant performed the task alongside 3 other individuals, all of whom were confederates of the experimenter. On arrival participants reported normal or corrected to normal vision, and they were asked to confirm their area of study, or department in which they work, at King’s College London. Following the experiment, participants were fully debriefed as to the true nature of the experiment. During a funnelled debrief – whereby participants were asked a series of questions which started broad and open-ended and funnelled to more specific questions to gauge their level of awareness of the true study aims – it was apparent that only one participant believed their fellow participants in the experiment to be confederates (and this one additional participant was excluded, leaving 21 participants’ data for analysis).

### 2.2 Experimental procedure

#### 2.2.1 Room and confederate setup

**Fig. 2:**
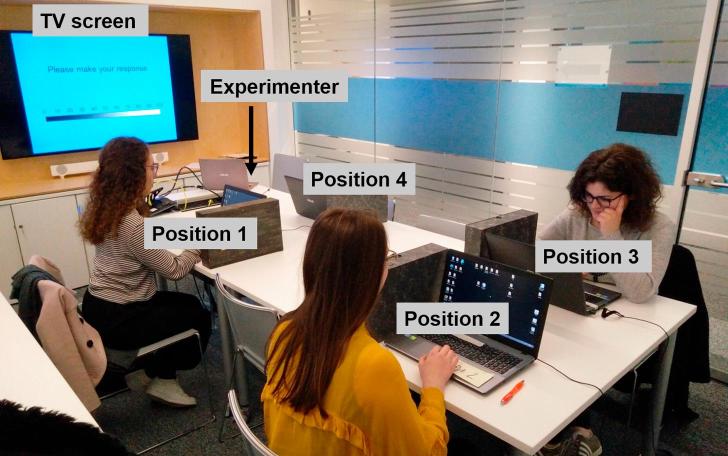
Room layout for the experiment showing locations of the main TV screen with respect to the experimenter and each of the 4 participant seating positions. All individuals appearing in the photo have given full permission for the use of this image.

The same 3 female confederates performed the task alongside each participant. They were undergraduate psychology students from King’s College London (mean age = 20.3 years), and their mean age did not differ significantly from the mean age of the participant sample (*p* > .05). In each instance, Participant 1 (confederate) arrived 15 min prior to the testing slot to ensure they were always first to arrive, taking a seat at Position 1 (see Fig. 2 for seating position labels). Following this, the real participant would be allowed time to arrive (most arriving a few minutes early for the testing session). The experimenter would instruct the participant to enter the testing room and take a seat at any laptop. To reduce uncontrolled interaction between the participant and confederates prior to the task, they were told to sit quietly whilst reading the instruction sheet and consent form. Due to the layout of the room, every participant without prompting chose to sit at Position 4. With the testing room door open to the half way point, Position 2 was blocked for the participant to take a seat in this position, and of the two positions remaining, seating themselves at Position 3 would block another participant’s access to Position 4. Thus, all participants followed the same pattern of seating themselves at Position 4 without direct instruction from the experimenter. Finally, in each instance, Participant 2 (confederate) would arrive a minute or two after the participant and take a seat at Position 2, followed by Participant 3 (confederate) who would arrive shortly after calling to ask for directions to the testing room and take the last remaining seat (Position 3). The participant number assigned to the confederates was counterbalanced across testing sessions. Finally, the 4 testing laptops (all 15.6 in., ASUS-Z550C, running Windows 10) were labelled with a sticker denoting the participant number prior to the testing session. See Fig. 2 for positions corresponding to the participant number assigned during the experiment.

#### 2.2.2 Visual perception task

**Fig. 3:**
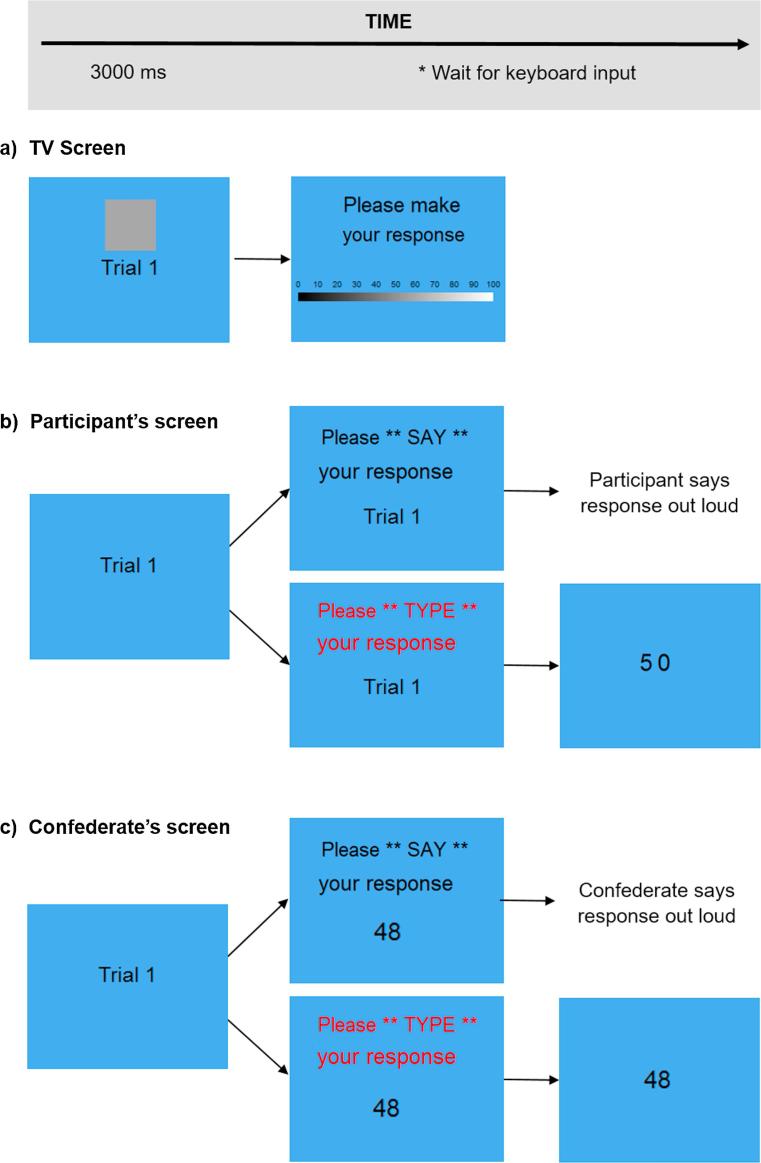
Example structure of one trial in the experiment showing display presented on (a) the main TV screen (visible to all participants), (b) the real participant’s screen and (c) a confederate’s screen. ^*^The TV screen programme waits for a space bar press to trigger the next trial, as do participant and confederate laptops during spoken trials. During typed trials, the programme waits for two digits to be typed before displaying the number on the screen, after which the programme waits for a space bar press to trigger the next trial.

The visual perception task was programmed and run using Cogent and Cogent Graphics for Matlab. In the task, participants were required to make judgements about the colour of squares presented on a large central TV screen (Samsung-DM65D, 65 in. display) running Windows 10 software; see Fig. 2 for location in the testing room). The colour of the squares could lie anywhere on a scale from white to black. Following the presentation of the square, a colour bar was presented on the TV screen. Participants were required to give a numerical response, matching the colour of the square with the same colour on the colour bar and reporting the colour’s numerical value (see Fig. 3). On some trials participants were asked to *type* their response using the numeric keyboard and on others they were asked to *say* their response out loud for the experimenter to write down. These instructions were given to each participant on their own laptop screen for each trial. They were instructed to pay very close attention to whether they should say or type their response on each trial, as this would sometimes differ across participants. For example, on any one trial, all participants could be required to *say* their responses out loud, or they may all be required to *type* their response. Alternatively, on some trials, one of the participants may be instructed to *type* their response whilst all other participants were instructed to *say* their response. Ostensibly in order that the experimenter could record the spoken responses by hand, participants were instructed to respond in ascending participant number order.

##### 2.2.2.1 Single trial structure

The experimenter verbally introduced each new trial and the trial number was displayed (e.g. ‘Trial 1’, ‘Trial 2’, ‘Trial 3’) on the main TV screen and each participant’s laptop screen. Each new trial (see Fig. 3 for a visual depiction of one trial) began with the trial number as well as a coloured square being presented on the main TV screen for 3 s. The trial number was also displayed on the participants’ laptop screens during these 3 s. The square was then replaced by a colour bar on the main TV screen (showing every shade from black to white; with labels in 10 step increments from 0 to 100), and simultaneously, each participant was instructed on their own laptop screen whether they should *type* or *say* their answer on that trial. Participant 1 then made their response, followed by the other participants in ascending participant order. On trials where any participant was instructed to type their response, their laptop would beep after two digits had been pressed using the numeric keyboard. This acted as an auditory cue for the next participant to respond. Participants were instructed they could respond using any number on the colour bar scale between 01 and 99. At the end of the trial, all participants and the experimenter pressed the space bar to move the experiment onto the next trial. The protocol began with 10 practice trials, followed by 153 trials in the main experiment, including a short break after each block of 51 trials. The task took approximately 45 min to complete.

##### 2.2.2.2 Trial type manipulations

To allow for public and private performance to be assessed within the same task, different trial types were introduced. First, to gauge the participants’ baseline performance when judging the colour of the squares, there were 18 ‘silent’ trials during which all participants were instructed to *type* their response. Second, ‘private’ trials were introduced (27 in total) during which the participant was required to *type* their response (and therefore this response remained private) whilst all other participants were instructed to *say* their response. All 4 participants (i.e. the real participant plus each of the three confederates) experienced their own set of 27 private trials where they were required to type their response. These trials constitute the private condition when the participant responded privately, and reduced majority trials when each of the confederates responded privately. Finally, during ‘public’ trials, all participants were instructed to *say* their response (27 in total). Although these trials constitute the public condition for analysis purposes it should be noted that on reduced majority trials the participant was required to make their response publicly after two responses from confederates, therefore these trials could be considered public trials with only two confederate responses rather than three. Based on the seminal studies of Asch, 1951, Asch, 1955, one may expect to also see a conformity effect on reduced majority trials, although of lesser magnitude than on the public trials with three confederate responses. Across the whole experiment, responses on 30% of trials for each participant were typed, and 70% spoken.

##### 2.2.2.3 Confederate congruency manipulation

In line with Asch’s original conformity studies (Asch, 1951, Asch, 1955, Asch, 1956), within each of the private and public conditions there was a ratio of 1:2 congruent to incongruent trials. Here, congruency refers to the relationship between the correct response and the responses given by the confederates. On congruent trials participants gave a correct answer, while on incongruent trials they gave an incorrect answer. Confederates were instructed as to which response to give via their laptop screens. During each trial, according to the trial type, a number was calculated for each confederate as a response by taking the correct square colour (e.g. 50) and adding or subtracting values from this. On an incongruent trial, 15 was added to or subtracted from this value (e.g. 35 or 65), as well as a small amount of jitter between 0 and 3 (e.g. values between 32 and 38 on a trial where the confederates responded lower than the correct response of 50, or between 62 and 68 on a trial where the confederates responded higher than the correct response of 50). On a congruent trial, however, only the jitter of 0, 1, 2, or 3 was added to or subtracted from the correct response (e.g. confederate responses could vary between 47 and 53 on a trial where the correct response was 50). This procedure was adopted in order to induce some slight variation between confederate responses to mask their status as confederates.

## 3 Results

For each trial a ‘response discrepancy’ was calculated as the difference between the participant’s response and the correct response. These values were calculated relative to the direction of the responses of the confederates. Thus, positive values indicate a discrepancy in the participant’s response in the direction of the confederates’ responses and negative values indicate a discrepancy in the opposite direction to that of the confederates. For example, for a trial where the correct response was 50 and confederates responded higher on the scale, a response of 55 by the participant would be considered a response discrepancy of +5, whereas a response of 45 would be considered a response discrepancy of −5. However, for a trial where the correct response was again 50 but confederates responded lower on the scale, a response of 55 would be a response discrepancy of −5, whilst a response of 45 would be a response discrepancy of +5. This allows for a calculation of the degree to which participants shift their responses towards or away from the confederates’ responses during private and public trials. The private and public conformity effects were calculated as the difference between response discrepancies during their respective congruent and incongruent trials, thus providing a measure of the informational influence effect (indexed by the size of the private effect) and the additional normative influence during public trials (indexed by the size of the public effect minus the private effect).

Trials for which response discrepancies were ±2.5 standard deviations from each participant’s mean for each condition were discarded with an a priori threshold of 15% lost trials for participant inclusion. As it was not necessary to discard more than 15% of data for any participant, all participants’ data were retained for full data analysis.

**Fig. 4:**
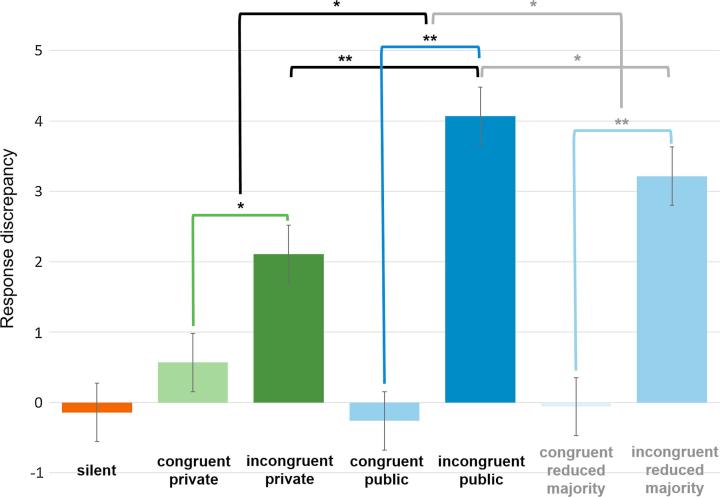
Bar graph representing mean response discrepancies for each trial type. ** indicates significance at *p* < .001 and * indicates significance at *p* < .05. Grey lines show comparisons between standard public trials and reduced majority public trials.

Fig. 4 displays mean response discrepancies during each trial type. As observed in the original Asch paradigm, baseline performance was excellent; the mean absolute response discrepancy from the correct response during silent trials was 1.42 (standard error of the mean [SEM] = 0.16). When signed rather than absolute scores were analysed, response discrepancies during silent trials did not significantly differ from 0 [*t*(20) = 0.40, *p* = .696]. Thus, participants could perform the task with a high degree of accuracy when they were not subject to responses of the confederates. Moreover, performance did not significantly differ from 0 during congruent trials for either private [*t*(20) = 1.69, *p* = .106] or public [*t*(20) = 0.76, *p* = .456] conditions. However, in line with our first prediction, conformity was observed towards the confederates’ responses in both public and private conditions, whereby response discrepancies were significantly larger on incongruent trials relative to congruent trials in both the private (private congruent response discrepancy = 0.57, SEM = 0.34, private incongruent = 2.10, SEM = 0.36; *t*(20) = 3.10, *p* = .006, *d* = 0.96) and public conditions (public congruent = −0.26, SEM = 0.34, public incongruent = 4.07, SEM = 0.41; *t*(20) = 7.79, *p* < .001, *d* = 2.51).

Data were entered into a repeated-measures two-way ANOVA, with condition (private or public) and congruency (congruent or incongruent) as within-subjects factors. This revealed no significant overall difference between performance in private and public trials [*F*(1, 20) = 3.67, *p* = .070, *η_p_*^2^ = .16], but a significant main effect of congruency [*F*(1, 20) = 69.76, *p* < .001, *η_p_*^2^ = .78] and crucially, a significant condition*congruency interaction [*F*(1, 20) = 12.66, *p* = .002, *η_p_*^2^ = .39], showing congruency (or conformity) effects to be significantly larger during public compared to private trials.

The conformity effect during private trials (private incongruent – private congruent response discrepancies) can be considered to arise from informational influence (i.e. *acceptance* of the group response) and this effect is significantly different from 0 [*t*(21) = 3.10, *p* = .006]. The additional conformity effect observed on public trials (public minus private effect) can be accounted for by normative influence (i.e. *compliance* to the group), and is also significantly different from 0 [*t*(21) = 3.56, *p* = .002]. Thus, in line with our second prediction, we see the presence of both informational and normative influence. Moreover, it is possible to compare the relative magnitude of informational influence (indexed by the private conformity effect; mean = 1.53, SEM = 0.50) and normative influence (indexed by public conformity minus private conformity effects; mean = 2.80, SEM = 0.79). The normative influence is numerically, but not statistically, significantly larger than that of informational influence [*t*(20) = 1.06, *p* = .303].

A significant congruency effect was also observed on public trials when responses of only two confederates were available to the participant [i.e. on reduced majority trials; *t*(20) = 9.05, *p* < .001, *d* = 2.73], whereby response discrepancies were larger during incongruent (mean = 3.21, SEM = 0.32) than congruent trials (mean = −0.59, SEM = 0.19). To examine our third prediction concerning the impact of the size of the majority (number of confederate responses available to the participant) on performance during public trials, we compared performance between public trials where responses of 2 versus 3 confederates were available to the participant. Response discrepancies during incongruent public trials were significantly larger during trials where the majority consisted of 3 confederates (mean = 4.07, SEM = 0.41) than 2 confederates (mean = 3.21, SEM = 0.32; *t*(20) = 2.72, *p* = .013, *d* = 0.51). Moreover, there was a significantly increased public congruency effect under a majority of 3 (mean = 4.33, SEM = 0.56) compared to 2 (mean = 3.27, SEM = 0.36; *t*(20) = 2.29, *p* = .033, *d* = 0.49) confederates.

Finally, the magnitude of informational and normative influence can be compared for trials in which only two confederates’ responses were available to the participant. Once again, the normative influence here (mean = 1.74, SEM = 0.63) is numerically, but not statistically, larger than that of informational influence (mean = 1.53, SEM = 0.50; *t*(20) = 0.19, *p* = .851). The normative influence arising from 3 confederate trials was significantly greater than the normative influence arising from 2 confederate trials [*t*(20) = 2.29, *p* = .033, *d* = 0.32].

## 4 Discussion

Compliance and acceptance are types of social influence which can be discriminated on the basis of their effect on the beliefs or attitude of the individual. Compliance occurs when the individual publicly accepts the group’s position but privately adheres to their own belief, while acceptance occurs when the individual internalises the belief or attitude of the group such that it becomes their own. Compliance is thought to be the result of normative influence; where the individual complies with group norms due to the social power of the group. Acceptance is thought to be the result of informational influence; where individuals seek information from others in order to determine the true state of the world. The impact of normative and informational influence can be determined through on-line conformity paradigms by comparing responses made by individuals in private and in public. Here we report a novel paradigm, based on that of Asch, 1951, Asch, 1955, in which both normative and informational influence can be measured on a within-subjects basis.

In line with the first prediction, as well as the original body of work by Asch, 1951, Asch, 1955, Asch, 1956, results indicated significant conformity effects both when participants’ responses were made in private and when made publicly in front of a group. With respect to the second prediction, it is thought that private conformity results solely from informational influence – acceptance of the group response without social pressure to conform – whereas public conformity results from both informational influence and normative influence – responding in line with the group in order to publicly comply with them. Crucially therefore, as has been reported in past comparisons of private and public conformity (Abrams et al., 1990, Asch, 1955, Deutsch and Gerard, 1955), a greater conformity effect was observed during public than private trials, demonstrating the presence of both informational influence in the private condition and the addition of normative influence during public conditions. Thus, we see demonstrations of both acceptance and compliance within the same task. Finally, results support the third prediction of increasing normative conformity with increasing size of the majority, replicating the finding by Asch (1955) whereby the size of the conformity effect increased as group size increased from 2 to 3.

The data reported here suggest that the paradigm is a valid test of social conformity. Based on Asch, 1951, Asch, 1955 original paradigm, it compared performance on a test of colour matching in a baseline condition when participants were unaware of the group’s judgements, when they were aware of the responses of three confederates but could give their own response in private, and when participants were aware of the responses of two or three confederates and had to give their responses in public. In common with Asch’s original findings, participants conformed to the judgements of others in the group; despite the task being sufficiently easy (participants’ responses were extremely accurate when not influenced by the group), responses were more inaccurate when the group gave incorrect responses. Also in common with the findings of Asch was the observation that the degree of conformity was greater when participants were exposed to three confederates than two. This was observed even though the ‘degree of conformity’ in the original Asch paradigm refers to the frequency of conformity, whereas here it refers to the magnitude of the conformity effect on a per trial basis.

The paradigm reported here has the advantage that measures of normative and informational influence are obtained from the same individual at the same point in time, facilitating sensitive comparisons as to their magnitude and enabling future studies using techniques such as fMRI which are reliant upon within-subject comparisons. In addition, the participant is not excluded from the group due to their private responses, avoiding the possibility of anti-conformity to the group judgements which may have influenced prior studies (Abrams et al., 1990). Finally, although limiting the direct comparison of effect sizes between the current paradigm and the Asch paradigm, the use of a continuous response scale allowed extremely subtle effects to be detected on a trial-by-trial and within-subject basis.

While the present results are encouraging, and support the validity of the task as a measure of conformity and its ability to measure informational and normative influence, it should be noted that the current study was primarily aimed at task development and validation. Therefore, variance due to key individual differences previously investigated with the Asch-style paradigm was deliberately reduced. For example, due to the literature on sex differences in social conformity (Cooper, 1979, Eagly et al., 1981, Larsen et al., 1979), both concerning the sex of the participant and also whether the group is of the same or opposite sex to that of the participant, the current study included only female participants and a group of female confederates. Moreover, we restricted our sample to young adults (aged 19–30 years) to reduce age-related individual differences. The sample size was also relatively small, likely providing an inexact estimate of the population effect size.

It can be seen then, that although these results support the use of the task to measure compliance and acceptance, types of social influence thought to arise from normative and informational influence respectively, further work is required to establish the replicability of these findings, and how they are moderated by factors such as age, sex, and culture.

## 5 Conclusions

Here we present data from a small sample validating a task designed to identify compliance and acceptance as a result of group influence, and therefore to identify normative and informational influences on decision-making. Although heavily-based on the conformity paradigm developed by Asch, 1951, Asch, 1955, the task has several advantages over previous versions of the Asch paradigm. First, public and private responding is manipulated on a within-subjects basis rather than between-subjects, providing a more sensitive measure of any difference in social influence in the two conditions. This feature also allows participants to respond in the same manner as confederates, reducing the likelihood that they will classify themselves as an out-group member. Second, participants were able to respond on a continuous scale – this enabled a graded measure of conformity to be established such that the magnitude of any effect on a single trial could be measured and subtle effects of conformity detected. Results supported previous claims of both normative and informational influences in Asch-type paradigms, with normative influence increasing with increasing majority size in such paradigms.
